# Sirtuin family in autoimmune diseases

**DOI:** 10.3389/fimmu.2023.1186231

**Published:** 2023-07-06

**Authors:** Zhengjie Tao, Zihan Jin, Jiabiao Wu, Gaojun Cai, Xiaolong Yu

**Affiliations:** ^1^ Science and Education Section, Wujin Hospital Affiliated with Jiangsu University, Changzhou, Jiangsu, China; ^2^ Department of Ultrasonics, The Wujin Clinical College of Xuzhou Medical University, Changzhou, Jiangsu, China; ^3^ Clinical Lab, Changzhou Second People’s Hospital Affiliated to Nanjing Medical University, Changzhou, China; ^4^ Department of Immunology, Wujin Hospital Affiliated with Jiangsu University, Changzhou, Jiangsu, China; ^5^ Cardiology, Wujin Hospital Affiliated with Jiangsu University, Changzhou, Jiangsu, China

**Keywords:** sirtuins, autoimmune diseases, immune inflammation, oxidative stress, treatment, review

## Abstract

In recent years, epigenetic modifications have been widely researched. As humans age, environmental and genetic factors may drive inflammation and immune responses by influencing the epigenome, which can lead to abnormal autoimmune responses in the body. Currently, an increasing number of studies have emphasized the important role of epigenetic modification in the progression of autoimmune diseases. Sirtuins (SIRTs) are class III nicotinamide adenine dinucleotide (NAD)-dependent histone deacetylases and SIRT-mediated deacetylation is an important epigenetic alteration. The SIRT family comprises seven protein members (namely, SIRT1–7). While the catalytic core domain contains amino acid residues that have remained stable throughout the entire evolutionary process, the N- and C-terminal regions are structurally divergent and contribute to differences in subcellular localization, enzymatic activity and substrate specificity. SIRT1 and SIRT2 are localized in the nucleus and cytoplasm. SIRT3, SIRT4, and SIRT5 are mitochondrial, and SIRT6 and SIRT7 are predominantly found in the nucleus. SIRTs are key regulators of various physiological processes such as cellular differentiation, apoptosis, metabolism, ageing, immune response, oxidative stress, and mitochondrial function. We discuss the association between SIRTs and common autoimmune diseases to facilitate the development of more effective therapeutic strategies.

## 1 Introduction

**Figure 1:**
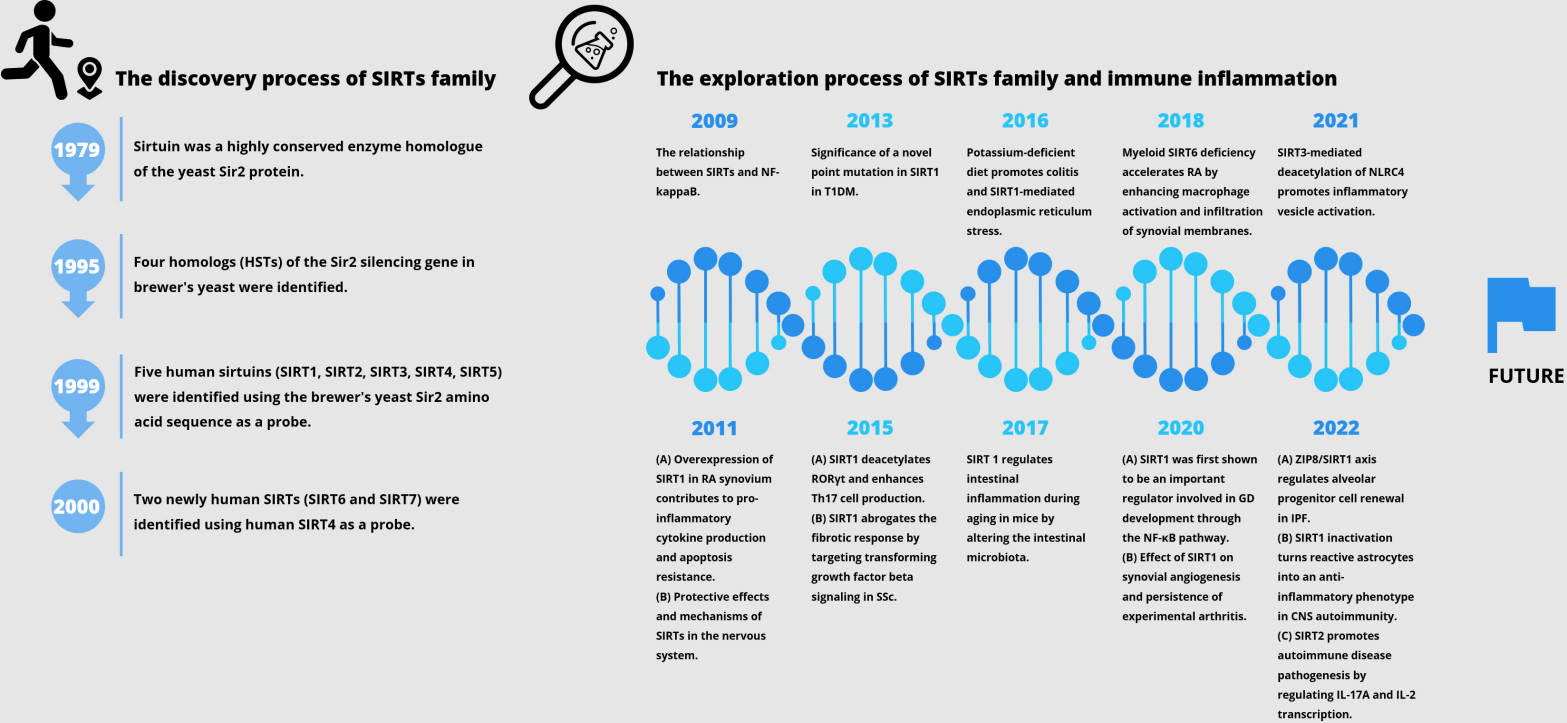
The history of the discovery of the SIRT family and important studies related to autoimmune diseases.

Histone deacetylase (HDAC) is an enzyme that catalyzes the removal of acetyl functional groups from lysine residues of histones and non-histones. At present, there are 18 known HDAC enzymes classified into four categories: Class I Rpd3-like proteins (HDAC1, HDAC2, HDAC3, and HDAC8); Class II Hda1-like proteins (HDAC4, HDAC5, HDAC6, HDAC7, HDAC9, and HDAC10); Class III Sir2 like proteins (SIRT1, SIRT2, SIRT3, SIRT4, SIRT5, SIRT6, and SIRT7); and class IV protein (HDAC11) (1). SIRTs (Sirtuins) are highly conserved enzyme homologues of the yeast Sir2 protein, originally identified as mate-type regulator 1 (MAR1) in Saccharomyces cerevisiae by Klar et al. in 1979, and the mutations at this locus lead to yeast transgenic failure (2). At the end of the 20th century, four homologues of Sir2 silencing genes had also been reported, and the Sir2 gene family was further investigated for its role in silencing, cell cycle progression, and chromosome stability (3). Sir2 has been reported to be associated with yeast longevity (4). Using the brewer’s yeast Sir2 amino acid sequence as a probe, Frye et al. identified five human SIRTs (namely, SIRT1, SIRT2, SIRT3, SIRT4, and SIRT5) and observed possible protein ADP-ribosyltransferase activity (5). As confirmed by Imai et al. in 2000, yeast and mouse Sir2 proteins are nicotinamide adenine dinucleotide (NAD)-dependent histone deacetylases associated with genome silencing and ageing (6). During the same period, similar identification was conducted using human SIRT4 as a probe, and two new human sirtuins (SIRT6 and SIRT7) were discovered (7). SIRTs have some similarities and differences compared to HDACs of categories I, II, and IV. Firstly, SIRTs are the only cofactors that require NAD as enzyme activity. NAD is used as a reactant to deacetylate the acetyllysine residues of the protein substrate that forms nicotinamide, and the deacetylated products and metabolite 2’-O-acetyl-ADP ribose, which is different from the zinc ion dependence catalyzed by other categories of enzymes. Secondly, HDAC types I, II, and IV belong to the arginase/deacetylase superfamily proteins, which contain arginase-like amide hydrolases and histone deacetylases. SIRTs exhibit single ADP ribosyltransferase and histone deacetylase activity. In addition, SIRTs have clearer intracellular localization. Finally, all four types of HDAC have both histone and non-histone substrates in eukaryotes. Herein, we review the history pertaining to the discovery of the SIRT family, as well as important studies related to autoimmune aspects (**Figure 1**).

## 2 Structure and function of SIRTs

Historically, seven mammalian homologues of yeast Sir2 (SIRT1 to SIRT7) have been identified, and all require cofactor NAD as a substrate. A common feature of SIRT family proteins is the conserved catalytic core region consisting of approximately 270 amino acids (8). The sequence between SIRT proteins is conserved and consistent, and the SIRT catalytic core region exhibits a high degree of structural overlap. The catalytic core region includes; (a) a large and structurally homologous Rosmann folding domain with NAD binding protein characteristics; (b) more diverse and smaller zinc-binding domains in the structure; (c) and loops that connect the Rosmann folding domain with the zinc-binding domain (9, 10). These loops form obvious extended cracks between the large and small domains, where NAD and peptide substrates containing acetyllysine enter from the opposite side and bind to the enzyme. The reaction groups of amino acids involved in catalysis and two binding substrate molecules are buried in protein tunnels in the gaps between the two structural domains (10). The homologous catalytic cores of the seven subtypes of the SIRT family catalyze through the same deacylation mechanism, which is considered the second commonality, including the following processes: (a) NAD and acetyllysine substrate binding; (b) Glycosidic bond cleavage; (c) Acetyl transfer; And the formation of (d) O-acetyl-ADPR, nicotinamide, and deacetylated lysine products (11).

The differences between SIRT family proteins were initially believed to be due to their different subcellular localization. Outside the catalytic core, SIRT proteins have variable N-and C-terminal regions. These regions are not conservative in the protein family, and their length, sequence and secondary structure differ. This will affect the subtype-specific localization and regulation of proteins (SIRT1 and 2 in the nucleus and cytoplasm; SIRT3, 4, and 5 in mitochondria; SIRT6 and 7 in the nucleus) (8). SIRT1 has the largest expansion in mammalian subtypes, including the N-terminal STAC binding domain (SBD) and inherent disordered regions (12, 13). Mitochondria SIRT3, 4, and 5 have very short extensions, especially the N-terminal mitochondrial localization sequence (MLS) (14). The catalytic activity level of members of the SIRT protein family is their second significant difference. Because the pocket of the active site containing the acyl group of the substrate shows remarkable difference among the subtypes, the subtype specificity of Lys acylation of different proteins is caused (8). Although there are slight differences in peptide binding slots between subtypes of the SIRT family, this has a relatively small impact on the catalytic activity of each subtype (15).

SIRT1, 2, and 3 show robust deacetylation activities. SIRT4 and SIRT6 display ADP-ribosyl transferase activity, used to transfer ADP-ribose from NAD to the substrate, thus yielding nicotinamide as a product (16). There are physical and functional interactions between SIRT4 and the components of pyruvate dehydrogenase complex (PDH). The lipase cofactor from the E2 component dihydroacyl lysine acetyltransferase (DLAT) is enzymatically hydrolyzed, which reduces the activity of PDH, thus affecting the glycolysis and tricarboxylic acid cycles (17). Additionally, SIRT6 prefers long-chain fatty acylations over acetylations due to a hydrophobic, wider acyl binding region than in other isoforms (18, 19). Free fatty acids can also bind and activate the deacetylation activity of SIRT6, which may explain the strong *in vivo* deacetylation activity of subtypes (20). SIRT5 exhibits enzyme activity mediated by specific Arg for malonylation, succinylation, and glutarylation (21, 22). SIRT7 shows deacetylation activity and is localized in nucleoli that govern the transcription of RNA polymerase I (23). Many targets can be modified by SIRTs, including histones and non-histones. SIRTs participate in a series of cellular metabolism and function regulation by modifying these targets, including inflammation, oxidative stress, mitochondrial function, immune response, and cellular differentiation, proliferation, and metabolism. Overall, the SIRT family plays significant roles in the regulation of cellular function and metabolism (**Table 1**). However, our current understanding of the SIRT protein family is limited, and it is necessary to conduct more research in this field.

**Table 1:** The structure and function of SIRTs.

SIRTs	Uniprot ID (From:http://www.uniprot.org/)	Organism	Length	Structure (From: AlphaFold)	Intracellular localizations	Enzyme activity	Targets	Functions
**SIRT1**	Q96EB6	Homo sapiens (Human)	747 AA	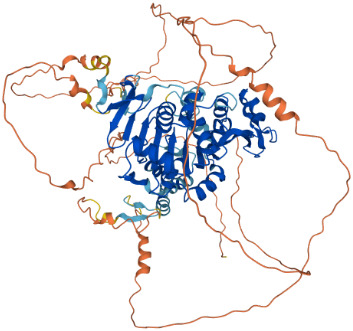	Nucleus/cytoplasm	Deacetylase	**Histones:** H1K26 H3K9,H3K14,H3K18,H3K56 H4K6,H4K12,H4K16 **Non-histones:** P53,FOXO1/3/4,HSF1,HIF1α,NF-κB,P300,TGF-β,PGC-1α	1.Cell metabolism 2.DNA transcription, repair 3.Inflammation inhibition 4.Oxidative stress
**SIRT2**	Q8IXJ6	Homo sapiens (Human)	389 AA	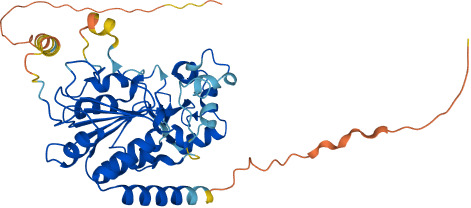	Nucleus/cytoplasm	Deacetylase	**Histones:** H3K56 H4K16 **Non-histones:** MnSOD2,p53,PGC1-α,CypD,FOXO3a,AMPK	1.Cell metabolism 2.DNA replication, transcription, translation 3.Cell cycle 4.Inflammation regulation
**SIRT3**	Q9NTG7	Homo sapiens (Human)	399 AA	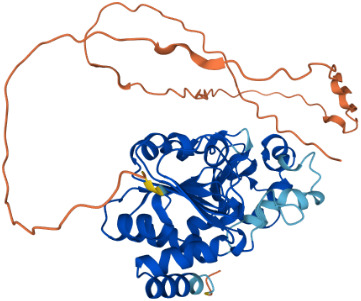	Nucleus/Mitochondria	Deacetylase	**Histones:** H3K56 H4K14 **Histones:** SOD2,PDMC1a,IDH2, GOT2,FoxO3a	1.Cellular metabolism 2.Inflammation inhibition 3.Oxidative stress 4.Apoptosis 5.Autophagy
**SIRT4**	Q9Y6E7	Homo sapiens (Human)	314 AA	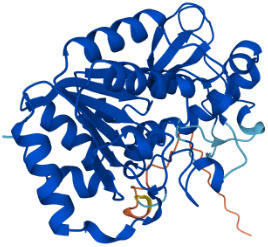	Mitochondria	1.ADP-ribosyltransferase 2.Lipoamidase 3.Deacetylase	**Non-histones:** GDH,PDH,ANT	1.Cellular metabolism 2.Mitochondrial metabolism 3.Oxidative stress
**SIRT5**	Q9NXA8	Homo sapiens (Human)	310 AA	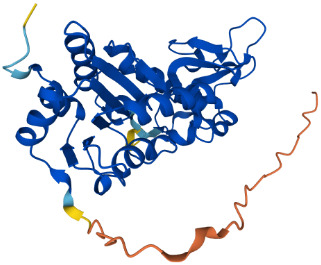	Mitochondria	1.Succinyl deacylase 2.Malonyl deacylase 3.Deacetylase	**Non-histones:** CPS1	1.Cellular metabolism 2.Immune regulation
**SIRT6**	Q8N6T7	Homo sapiens (Human)	355 AA	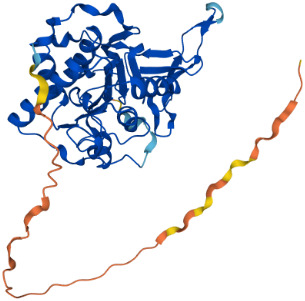	Nucleus	1.Deacetylase 2.ADP-ribosyltransferase 3.Long-chain fatty acyl deacylase	**Histones:** H2BK12, H3K9,H3K17,H3K18,H3K27,H3K56 **Non-histones:** PGC1-α,TRF2,KAP1,TNF-α	1.Cell metabolism 2.Chromatin and DNA repair
**SIRT7**	Q9NRC8	Homo sapiens (Human)	400 AA	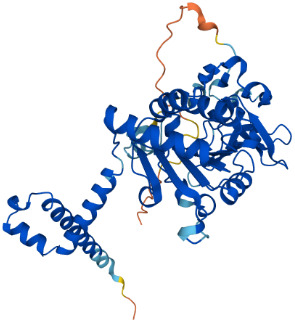	Nucleus	Deacetylase	**Histones:** H3K18 **Non-histones:** Hif-1α,Hif-2α,EIA,Smad6	1.Gene transcriptional regulation 2.Chromatin and DNA repair

## 3 Relationship between SIRTs and immune inflammation

**Figure 2:**
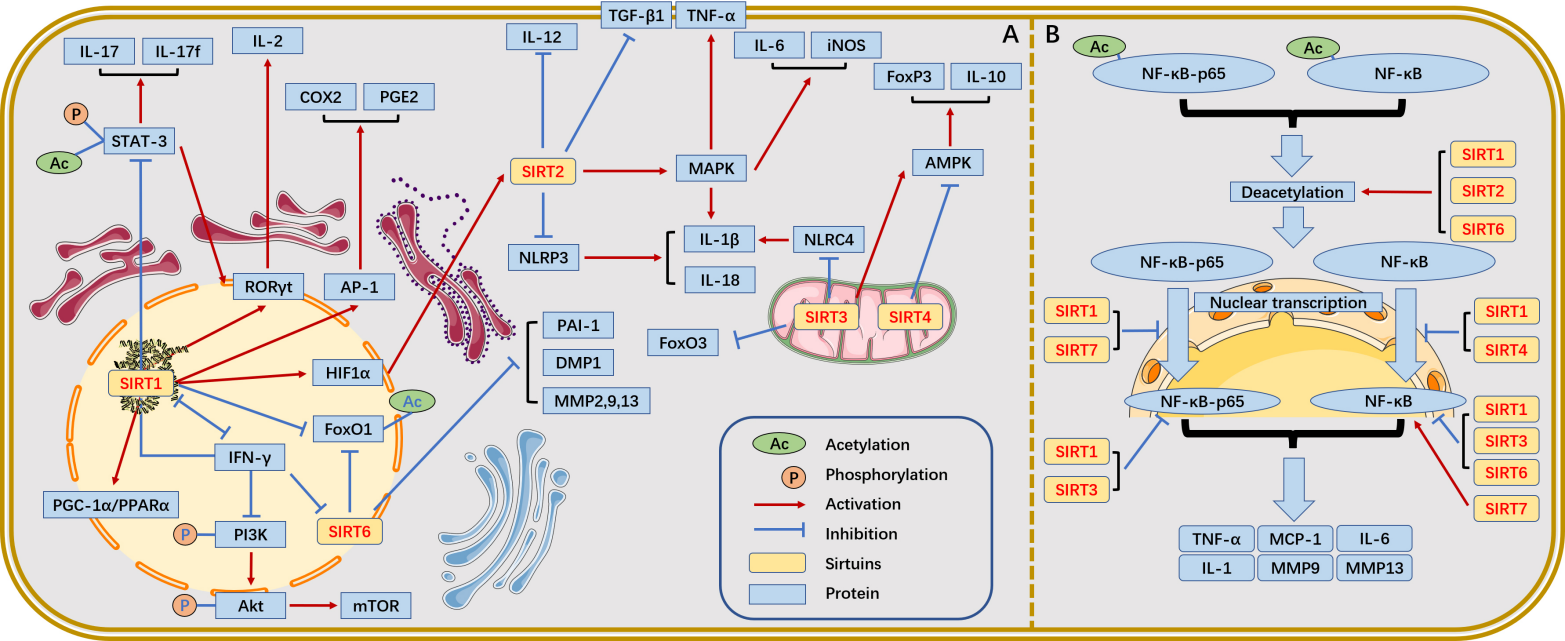
The relationship between SIRTs and immune inflammation. **(A)** Relationship between SIRTs and immune inflammation. **(B)** The most widely studied NF-κB pathway in the SIRT family and the different roles of SIRTs in this process.

SIRTs, to some extent, influence autoimmune disease progression by epigenetically modifying the targets affecting immune cells and immune responses. We summarize the relationship between SIRT family and immune inflammation (**Figure 2**).

In the nuclear factor-κB (NF-κB) pathway, SIRT1 binds to and deacetylates c-Fos and c-Jun, thereby inhibiting activator protein-1 (AP-1) transcriptional activity and reducing AP-1-associated cyclooxygenase-2 (COX2) and prostaglandin (E2) expression in peritoneal macrophages (24). However, deletion of SIRT1 would lead to increased tumour necrosis factor-α (TNF-α) and interleukin (IL)-1β levels by hyperacetylation of NF-κB p65 in macrophages (25, 26). Additionally, blockade of the AP-1 signalling pathway inhibits T cell activation and proliferation. SIRT1 regulates the production of cytokines (e.g., IL-12, TGFβ-1) *via* HIF-1α in dendritic cells (DCs) while inhibiting Th1 and promoting Treg differentiation (27). SIRT1 is expressed at high levels in intestinal inflammatory Th17 cells and deacetylates not only retinoic acid receptor-related orphan receptor-γt (RORγt), which activates IL-17 and inhibits the IL-2 promoter to stimulate Th17 cell differentiation but also STAT3, which prevents Th17 differentiation under certain circumstances (28, 29). In the NF-κB pathway, SIRT2 is present in a complex with p65 when the cell is unstimulated. Upon TNFα stimulation, p65 translocates to the nucleus, while SIRT2 remains in the cytoplasm. Coactivator p300 binds to nucleus p65 and is acetylated at K310, K314 and K315 to fine-tune gene expression. When the NF-κB response is terminated, p65 shuttles back to the cytoplasm, and SIRT2 deacetylates p310 at K65, thereby resetting the entire NF-κB response (30). Thus, SIRT2 can reduce the expression of NF-κB-dependent genes, IL-1β, IL-6, monocyte chemotactic protein-1, matrix metalloproteinase (MMP)-9 and MMP-13, thereby exerting anti-inflammatory effects (31).In the NLRP3 inflammatory signalling pathway, targeted NLRP3 deacetylation by SIRT2 inhibits the assembly and activation of NLRP3 inflammatory vesicles, leading to the attenuation of IL-1β- and IL-18d-induced inflammatory response (32). In the mitogen-activated protein kinase (MAPK)-related inflammatory signalling pathway, SIRT2 deacetylates MKP-1, decreases the acetylation level of MKP-1, promotes Toll-like receptor signalling, and upregulates MAPK signalling, thereby promoting the production of proinflammatory factors IL-1β, IL-6, TNF-α, and iNOS and eventually leading to inflammation (33, 34).

The activation of SIRT3 deacetylase activity by SENP1 in the mitochondria of T cells promotes T cell survival and memory T cell development, which are mediated by 5’-AMP-activated protein kinase (AMPK) (35). SIRT3 deficiency activates the deacetylation at NLRC4 inflammatory vesicles Lys71 and Lys272, which mediate IL-1β production (36). SIRT4 overexpression blocks Treg cell production by conventional T cells *in vitro*, whereas SIRT4 knockdown has been shown to enhance the anti-inflammatory activity of Tregs in the injured spinal cord parenchyma of mice. In Treg cells, SIRT4 downregulates the expression of AMPK, FOXP3, IL-10, and TGF-β; conversely, the AMPK agonist AICAR restores the expression of FOXP3 and IL-10 in SIRT4-overexpressing Treg cells (37). SIRT5-deficient mice are prone to dextran sulfate sodium (DSS)-induced colitis, which is associated with high PKM2 succinylation and IL-1β production due to SIRT5 deficiency (38). SIRT5 deficiency induces stronger T cell activation, as evidenced by an imbalance in the differentiation subpopulations of Tregs and Th1 cells (39). Additionally, SIRT5 competes with SIRT2 to block p65 deacetylation by SIRT2 in a manner unrelated to deacetylase activity, leading to increased acetylation of p65 and activation of the NF-κB pathway and its downstream cytokines, thereby enhancing the innate inflammatory response of macrophages (40).

The unique regulatory role of SIRT6 in inflammatory diseases mainly depends on the activity of TNF-α and NF-κB as well as on several other factors within the regulatory functions of TNF-α and NF-κB. SIRT6 acts as a lysine deacylase that catalyzes the hydrolysis and secretion of TNF-α from cells, thereby functioning as a pro-inflammatory agent (19). Furthermore, SIRT6 interacts with Lys310 of the NF-κB p65 subunit and inhibits the expression of the proinflammatory factor NF-κB (41). Given that SIRT7 is involved in various immune-mediated inflammatory responses (including the NF-kB inflammatory pathway), SIRT7 may, therefore, be closely associated with intestinal immune-mediated inflammatory responses, such as inflammatory bowel disease (IBD). Kim et al. showed that SIRT7 attenuated colonic mucosal inflammation in mice (42).

Overall, there are many studies on the mechanisms of SIRTs family involvement in inflammation, especially involving NF-κB, TNF-α, NLRP3, and MAPK-related pathways. It is also worth noting that the effects of various SIRTs on disease may vary from disease to disease or even have opposite effects, as will be discussed later. Further studies are needed in the future to explore whether changes in the levels of SIRTs are common pathological changes in the onset and progression of inflammation-related diseases. Further attention is also needed to resolve some of the conflicting data and to better understand the key role of the SIRTs family in the inflammatory response. Here, we speculate that the conflicting roles of the SIRTs family in inflammation may be due to their regulation of common signalling pathways under specific pathological conditions. In summary, there is still a wide scope for research on SIRTs that needs to be further explored to determine the role of the SIRTs family in the inflammatory response and the potential mechanisms of action.

## 4 Relationship between SIRTs and oxidative stress

**Figure 3:**
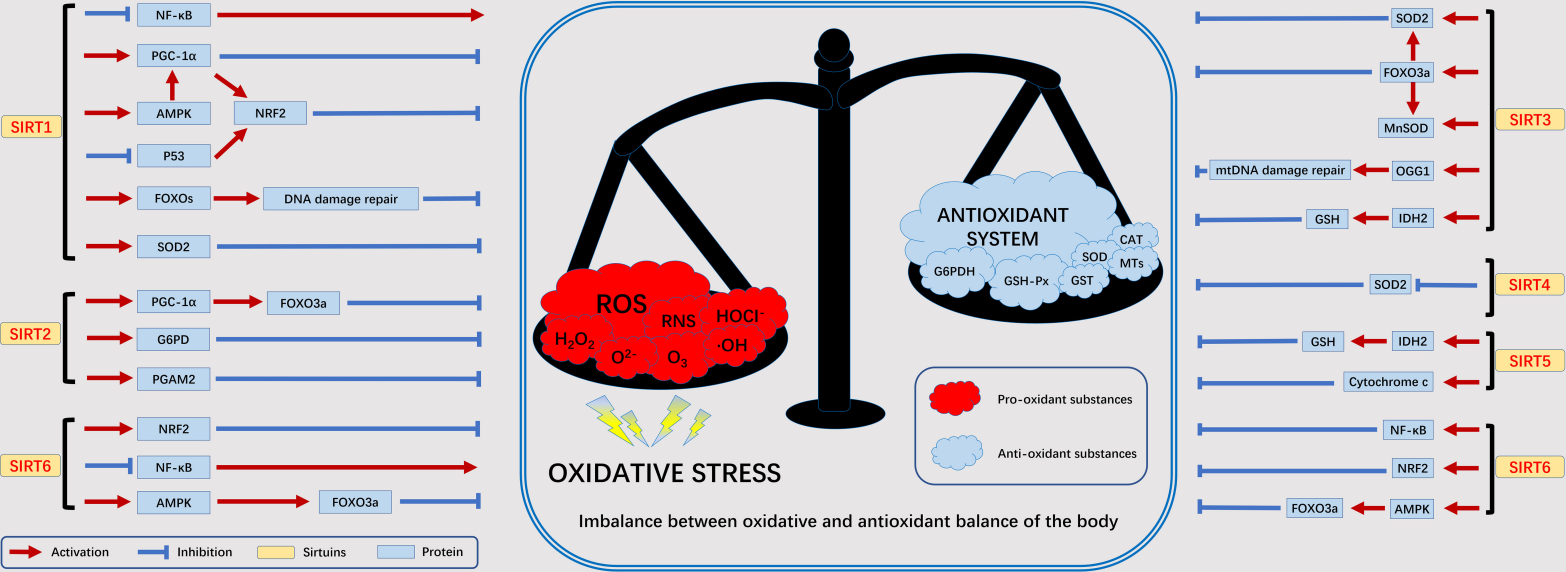
The relationship between SIRTs and oxidative stress. Excessive ROS produced by various exogenous as well as abnormal endogenous metabolic processes will lead to an imbalance in the oxidative and antioxidant balance of the body, in which the SIRT family is mainly involved in the antioxidant process. adenosine 5´-monophosphate (AMP)-activated protein kinase (AMPK), forkhead box protein O3a (FOXO3a), glucose 6-phosphate dehydrogenase (G6PD), glutathione SH (GSH), recombinant isocitrate dehydrogenase 2, mitochondrial (IDH2), manganese superoxide dismutase (MnSOD), nuclear factor kappa B subunit (NF-κB), nuclear erythroid 2-related factor 2 (NRF2), oxoguanine glycosylase 1 (OGG1), peroxisome proliferators-activated receptor γ coactivator 1alpha (PGC-1α), phosphoglycerate mutase 2 (PGAM2), superoxide dismutase 2 (SOD2).

Under normal conditions, nutrients in the body (e.g., glucose, fatty acids, etc.) are oxidized to release energy. As part of β-oxidation, glycolysis, and the Krebs cycle, this energy is transmitted *via* NAD by reduction to NADH (43). As we age, abnormal biochemical reactions of the body as well as pathophysiological processes will lead to the production of ROS. The SIRT family is NAD-dependent and can participate in the oxidative stress process in the body. The current study found that the SIRT family is involved in oxidative stress mainly associated with the following proteins or genes: NF-κB, NRF2, FOXOs, PGC-1α, p53, and AMPK (11). We summarize here the relationship between the SIRT family and oxidative stress-related factors to facilitate our later discussion of the disease (**Figure 3**).

NRF2 plays an extremely important role in antioxidant response element (ARE)-dependent transcriptional regulation. NRF2 regulates the expression of antioxidant genes by mutating upon stimulation and interacting with ARE (44). SIRT1 activates Nrf1 by altering the structure of Keap2, leading to NRF2 nuclear translocation and promoting the expression of antioxidant genes, such as glutathione transferase (45). In peripheral nerve injury, downregulation of spinal SIRT2 inhibits NRF2 activity leading to oxidative stress (46). Overexpression of SIRT6 enhances NRF2 signalling to reduce oxidative stress in brain tissue (47). In the FOXOs family, FOXO1, FOXO3 is involved in important oxidative stress processes by regulating the scavenging of excess ROS by downstream target genes such as upregulated manganese superoxide dismutase (MnSOD) and catalase (CAT). SIRT1 induces FOXO1 translocation and increases the level of FOXO1 protein in adipocytes, thereby reducing ROS and oxidative stress production (48). In addition, SIRT3 also activates FOXO3 gene expression to resist oxidative stress (49). PGC-1α can scavenge excessive ROS, induce antioxidant enzyme expression and maintain mitochondrial function to block oxidative stress injury. SIRT1 can activate PGC-1α through deacetylation to scavenge oxidative stress-induced ROS and alleviate oxidative stress injury (50). In addition, PGC-1α and SIRT3 can directly interact with each other to exert antioxidant capacity (51). p53 as a regulator can play a role in promoting oxidative stress and antioxidant. It promotes oxidative stress injury by regulating targets such as glutathione/NADH. Inhibit oxidative stress by regulating MnSOD, glutathione peroxidase 1 and Jun N-terminal kinase (JNK) (52). SRT2104 (SIRT1 agonist) was used to enhance the expression and activity of renal SIRT1 and deacetylate p53, leading to the activation of antioxidant signalling (53). AMPK is a major regulator of metabolic homeostasis and is activated under conditions of oxidative stress. Overexpression of SIRT1 leads to deacetylation of liver kinase B1 (LKB1), an upstream regulator of AMPK, which activates AMPK to attenuate oxidative stress (54). In addition, SIRT6 promotes AMPK expression and upregulates gene expression of MnSOD and CAT proteins, thereby inhibiting oxidative stress (55).

In addition, oxidative damage induces the formation of large silencing complexes containing DNA methyltransferases and constituents of the polycomb (56). This damage induces the recruitment of key components of the complex from transcriptionally depleted regions of the genome to GC-rich regions, including the promoter CpG island. the components of PRC4, SIRT1 and EZH2, are tightly bound to chromatin. Due to the high amount of guanine enriched in GC-rich regions, which are the most easily oxidized of the four deoxyribonucleosides, they may be the primary targets of oxidative damage (57). This oxidative stress-induced translocation leads to changes in histone labelling, transcription and DNA methylation, resulting in gene silencing in regions of DNA damage. Thus it can promote genome stability.

In conclusion, as shown in **Figure 3**, the maintenance of redox homeostasis is achieved by balancing mechanisms acting at various complex levels associated with transcriptional regulation. These studies reflect the importance of the SIRTs protein family in oxidative stress, acting synergistically through different mechanisms to enhance intracellular homeostasis. The apparent protective effect of SIRTs against oxidative stress could serve as a mechanistic basis for the development of antioxidants and modulators.

## 5 Role of SIRTs in organ-specific autoimmune diseases

### 5.1 Endocrine system: Graves’ disease, Hashimoto’s thyroiditis, Type 1 diabetes mellitus

Little has been reported regarding the role of SIRT1 in Graves’ disease, a classic autoimmune thyroid disease. Sarumaru et al. showed that polymorphisms in the SIRT1 gene were associated with an increase in thyroid autoantibody production (58). SIRT1 may act as a negative regulator of GD-related inflammatory processes. Compared with healthy controls, patients with Graves’ disease exhibited reduced SIRT1 expression and activated NF-κB pathway in their peripheral blood. Further analysis revealed that SIRT1 inhibited the NF-κB pathway activity *via* p65 deacetylation (59). But there are also reports to the contrary that dysregulation of epigenetic modification genes in peripheral blood mononuclear cells (PBMCs) in patients with Graves’ disease led to a significant increase in the mRNA expression of histone H4 hypoacetylation and histone deacetylases 1 and 2 (60). The difference between the two is unclear for now. As in other autoimmune diseases, Treg function is defective in Graves’ disease. By using a flow cytometry assay and conducting a protein blotting analysis, Zhang et al. detected more IL-17^+^ T cells in parallel with a decrease in Treg cells. FOXP3 and miR-23a-3p were significantly downregulated in patients with Graves’ disease, whereas SIRT1 and RORγt were upregulated. Further studies showed that the aberrant acetylation of FOXP3, which is regulated by miR-23a-3p by targeting SIRT1, mediated the defective Treg function in patients with Graves’ disease (61).

Hashimoto’s thyroiditis is similar to Graves’ disease in that reduced FOXP3 expression levels, and defective Treg function are regulated by SIRT1-mediated aberrant FOXP3 acetylation in patients with Hashimoto’s thyroiditis. Ex-527 (a SIRT1 inhibitor) upregulates the FOXP3 acetylation levels and subsequently increases the number of Treg cells and inhibits function (62). Additionally, oxidative stress interferes with the normal function of thyroid cells. Th1 cytokines drive oxidative stress and cause a significant decrease in SIRT1 expression associated with HIF-1α, GLUT-1, and VEGF-A upregulation, suggesting that SIRT1 (a key regulator of oxidative stress) may be regarded as a potential therapeutic target for Hashimoto’s thyroiditis (63).

SIRT1 plays a crucial role in Type 1 diabetes mellitus (T1DM), which results from autoimmune-mediated β-cell destruction, leading to insulin deficiency. Destruction and apoptosis of pancreatic β-cells are the typical features of T1DM. Biason-Lauber et al. identified a T–C exchange in exon 1 of SIRT1 corresponding to a leucine-proline mutation in residue 107. SIRT1-L107P expression in pancreatic islet β-cells leads to an overproduction of nitric oxide, cytokines, and chemokines (64). This mutation is likely to contribute to oxidative stress as well as inflammation that destroys pancreatic islet β-cells. Previous studies revealed that the application of curcumin (an anti-inflammatory, antioxidant, and anti-apoptotic substance) and tropisetron (a 5-HT3 receptor antagonist) led to a reduction in β-cell apoptosis and that both oxidative stress and endoplasmic reticulum stress played a key role in the apoptotic process. Excess ROS generated by stress, as well as inflammatory mediators, caused damage to the islet β-cells. However, curcumin inhibited the SIRT1/PERK/CHOP pathway to reduce apoptosis, whereas tropisetron attenuated inflammation by inhibiting SIRT1/NF-κB signalling (65, 66). Additionally, resveratrol reduced hyperglycemia-induced superoxide production by upregulating SIRT1, inducing FOXO3a, and inhibiting p47phox in monocytes, resulting in a protective effect against cellular oxidative stress (67).

### 5.2 Respiratory system: Pulmonary fibrosis

Pulmonary fibrosis is an irreversible interstitial pulmonary complication characterized by progressive exacerbation and an unpredictable clinical course. Recent developments in the field of pulmonary fibrosis indicate the critical role of SIRTs as potential anti-fibrotic drug targets in regulating disease progression. In particular, SIRT1, SIRT3, SIRT6, and SIRT7 have been recognized as protective SIRTs associated with pulmonary and metabolic diseases, including fibrosis (68).

Liang et al. reported that SIRT1 activation promoted ACE2 self-renewal and differentiation in patients with idiopathic pulmonary fibrosis (IPF) and aged mice by regulating the zinc transporter protein SLC39A8 (ZIP8), thereby attenuating pulmonary fibrosis (69). Additionally, Qian et al. showed that the expression of non-coding RNA sirt1 antisense (sirt1 AS) was significantly reduced in bleomycin (BLM)-induced pulmonary fibrosis and that sirt1 AS effectively inhibited the TGF-β1-modified epithelial-to-mesenchymal transition (EMT) *in vitro* and alleviated the progression of IPF *in vivo* (70). The two studies mentioned above show that increased SIRT1 promotes ACE2 and inhibits EMT action, both of which ultimately alleviate IPF. In addition, SIRT1 activation or overexpression can also attenuate pulmonary fibrosis *via* regulation of TGF-β1/p300 signalling (71). However, Zeng et al. observed that SIRT1 expression was significantly increased in the lungs of patients with IPF and a mouse model of BLM-induced pulmonary fibrosis. This seems contradictory to some extent, and further studies are necessary to clarify this, considering that SIRT1 expression in pulmonary fibrosis remains controversial.

Mesenchymal stem cell (MSC)-based therapy has emerged as a new strategy for treating IPF. It can exert benefits by affecting SIRT1-related pathways. Shi et al. observed that the expression of miR-199a-5p was significantly enhanced in the sera of patients with IPF and in IPF-MSCs, inducing the senescence of MSCs. miR-199a-5p inhibition ameliorated this process; mechanistically, miR-155-5p inhibition promoted autophagy and ameliorated the senescence of IPF-MSCs by activating the SIRT1/AMPK signalling pathway. Therefore, the transplantation of anti-miR-199a-5p-IPF-MSCs is expected to become a new therapeutic target in the future (72).

A previous investigation on the role of SIRT2 in pulmonary fibrosis revealed that SIRT2 expression was upregulated in human embryonic lung fibroblasts treated with TGF-β1. Treatment with the SIRT2 inhibitor AGK2 significantly attenuated the degree of pulmonary fibrosis and reduced the phosphorylation of Smad2/3; therefore, SIRT2 may be involved in IPF development by regulating the Smad2/3 pathway (73). SIRT3 is a mitochondrial protein deacetylase that regulates antioxidant response and mitochondrial homeostasis. Its absence may lead to alveolar epithelial cell injury and fibroblast-myofibroblast differentiation. Sosulski et al. showed that SIRT3 deficiency in the ageing mouse lung promoted TGF-β1-mediated fibrotic responses and increased Smad3 levels, possibly interacting with SIRT2 (74). SIRT3 deficiency promotes pulmonary fibrosis by enhancing mitochondrial DNA damage and apoptosis in alveolar epithelial cells. Cryptotanshinone has been suggested to be an effective treatment for pulmonary fibrosis, as it modulates the TGF-β1/Smad3, STAT3, and SIRT3 pathways (75). Restoration of SIRT3 gene expression through airway delivery has also been shown in mice to address age-related persistent pulmonary fibrosis (76). SIRT6 is involved in the TGF-β1 signalling pathway and inhibits alveolar cell fibrosis. Chen et al. observed EMT inhibition resulting from the inactivation of the TGF-β1/Smad2 signalling pathway (77). Additionally, SIRT6 inhibits the EMT in IPF by inactivating TGF-β1/Smad3 signalling, which emphasizes the critical role of SIRT6 in IPF (78). SIRT6 inhibits the NF-κB signalling pathway to block the pulmonary myofibroblast differentiation induced by TGF-β1 (79). Patients with pulmonary fibrosis have been reported to display the lowest SIRT7 expression levels in fibroblasts (80). The decrease in SIRT7 expression has a pro-fibrotic effect, which is mediated by changes in Smad3 levels. SIRT7 also regulates TGF-β-induced lung fibrosis *via* glutaminase 1 (81).

The above evidence suggests that SIRT1, SIRT3, SIRT6, and SIRT7 contribute to the prevention and amelioration of LPF pathogenesis and that the number of studies on SIRT2 remains insufficient, with the regulatory roles of other SIRT members being unclear. Mazumder et al. reviewed the regulatory roles of SIRTs in cellular and mitochondrial metabolic pathways that are critical for pulmonary fibrosis. They concluded that most SIRTs are protective against pulmonary fibrosis, except SIRT2, which may play a pro-fibrotic role given the pro-inflammatory effects observed in asthma (68). The potential role of SIRTs in regulating pulmonary fibrosis warrants further studies.

### 5.3 Digestive system: Inflammatory bowel disease

IBD is characterized by recurrent chronic intestinal inflammation and gastrointestinal bleeding and can be divided into two main types—namely, Crohn’s disease and ulcerative colitis. In recent decades, IBD has received increasing attention owing to its increasing incidence worldwide.

Some clinical studies have shown that the reduced activity of SIRT1 in various IBD models may contribute to the development of IBD. Caruso et al. detected insignificant SIRT1 RNA and protein expression in the lamina propria mononuclear cells from patients with IBD and observed that SIRT1 downregulation promoted the sustained production of inflammatory cytokines and oxidative stress in colitis. Subsequent treatment with the specific SIRT1 activator Cay10591 for IBD in lamina propria mononuclear cells decreased the NF-κB activation and inhibited the synthesis of inflammatory cytokines, whereas treatment with the SIRT1 inhibitor Ex-527 increased the release of interferon (IFN)-γ (82). Thus, exogenous administration of SIRT1 activators reduced colitis (83). Similarly, Ren et al. used the SIRT1 activator SRT1720 to reduce intestinal epithelial cell apoptosis in ulcerative colitis *via* inhibition of CHOP and cystein-12 (molecules associated with endoplasmic reticulum stress-mediated apoptosis); in contrast, administration of nicotinamide (a SIRT1 inhibitor) exerted the opposite effect (84). Additionally, resveratrol treatment had been shown to significantly improve DSS-induced colitis and restore the SIRT1-mRNA levels (85), to upregulate the expression of phosphorylated mammalian target of rapamycin (mTOR) and SIRT1 in colonic tissues, to reduce autophagy, and to regulate SIRT1/mTOR signalling *via* inhibition of the intestinal inflammatory cascade response (86). It is interesting to note that SIRT1 knockdown has also been confirmed to have a protective effect against IBD (83). SIRT1 gene deletion may help in maintaining gastrointestinal immune homeostasis to improve the disease status of colitis. Wellman et al. reported that intestinal epithelial SIRT1 regulated the intestinal microbiota (87). Furthermore, it improves the intestinal antibacterial defence to prevent intestinal inflammation (88). Thus, SIRT1 deletion in the intestine has a positive impact on IBD development. Akimova et al. investigated the role of SIRT1 targeting FOXP3^+^ T cells in chronic colitis in mice and showed that FOXP3^+^ T cells translocation in B6/Rag1 mice led to chronic colitis. Additionally, the secondary transfer of TE cells lacking Sirt1 to B6/Rag1^(-/-)^ mice resulted in a nearly three-fold increase in iTreg formation compared to that in wild-type TE cell-receiving mice. In the absence of SIRT1, naive T cells tended to differentiate into Tregs. In addition, treatment with EX-527 reduced weight loss and colonic inflammation but increased iTreg differentiation (89). Dong et al. showed that the protein kinase CK2 downregulated SIRT1 expression, was involved in Th17 inhibition, and promoted Treg differentiation (90). SIRT1 deficiency may inhibit colitis development by inducing Tregs.

In summary, both the activation and inhibition of SIRT1 exert a protective effect in patients with IBD. SIRT1 upregulation decreases NF-κB acetylation, leading to increased pro-inflammatory cytokine expression. In contrast, SIRT1 deletion or silencing suppresses colitis and maintains gastrointestinal homeostasis by inducing Tregs.

### 5.4 Nervous system: Multiple sclerosis

Multiple sclerosis is a neurodegenerative disease characterized by chronic inflammation of the central nervous system, in which several factors influencing disease susceptibility and progression act together. SIRT1 induces chromatin silencing *via* histone deacetylation and regulates cell survival by modulating transcriptional activity. A large number of cells express SIRT1 in both acute and chronic active multiple sclerosis, whereas SIRT1-mRNA and protein expressions are significantly reduced in PBMCs during relapse (91). The expression of SIRT1 correlates with acetylation and methylation of H3K9. Ciriello et al. collected PBMCs from patients with multiple sclerosis treated with glatiramer acetate to determine phosphorylated SIRT1 (p-SIRT1) and H3K9me3 levels. Compared to stable patients with multiple sclerosis, this relapse during which p-SIRT1 protein and H3K9me3 levels showed statistically significant differences (92). Therefore, SIRT1 may serve as a biomarker of relapse. Studies using animal models of demyelinating and neurodegenerative diseases have shown that SIRT1 induction ameliorates the disease process (93). Recent studies have reported that the loss and dysfunction of mitochondria and peroxisomes contribute to myelin and axonal damage in multiple sclerosis. Singh et al. observed that treatment for EAE with a combination of lovastatin and AMPK activator (AICAR), lovastatin-mediated RhoA inhibition, and AICAR-mediated AMPK activation in mice synergistically enhanced the expression of transcription factors and regulators required for biogenesis (e.g., PPARα/β, SIRT1), as well as mitochondrial and peroxisomal functions, providing a protective effect (94). Methylene blue (MB) treatment significantly reduced the clinical scores of experimental autoimmune encephalomyelitis (EAE) and attenuated pathological damage to the spinal cord, which was associated with activation of the AMPK/SIRT1 signalling pathway and inhibition of pro-inflammatory T cell responses (95, 96). Oral treatment with the SIRT1-activating compound SRTAW04 significantly increased SIRT1 activity within the optic nerve and prevented optic nerve meridian loss. SRTAW04 treatment significantly reduced ROS levels, increased mitochondrial function-related enzyme expression, and reduced demyelination, exerting a similar protective effect (97). In addition, Sirt3 and mitochondrial abnormalities may be associated with excessive fatigue or muscle dysfunction in multiple sclerosis. The use of ellagic acid (EA) was able to increase SIRT3 expression and reduce oxidative stress in muscle tissue, ultimately restoring mitochondrial function (98).

Immune cells are involved in regulating the expression of SIRTs and the pathogenesis of multiple sclerosis, but they are poorly studied. Th17 cell is an important component of the adaptive immune system and is involved in the pathogenesis of most autoimmune and inflammatory syndromes. SIRT1 increases the transcriptional activity of RORγt, the hallmark transcription factor of Th17 cells, and enhances Th17 cell production and function. Both T cell-specific SIRT1 deficiency and pharmacological SIRT1 inhibitor treatment inhibit Th17 differentiation and are protective in a mouse model of multiple sclerosis (28). For example, MB treatment reduces Th17 responses and increases Treg responses (95). *In vitro* and *in vivo*, lipocalin (ADN) upregulates SIRT1 and peroxisome proliferator-activated receptor γ (PPARγ) and inhibits RORγt, which can inhibit Th1 and Th17 and their cytokines *in vitro* (99). ADN deficiency mainly promotes antigen-specific Th17 cell responses in EAE. These results systematically reveal the effect of ADN on pathogenic Th17 cells and the underlying mechanism. B cells act as APCs to overactivate T cells and exacerbate the progression of multiple sclerosis. MiR-132 overexpression in B cells significantly enhanced lymphotoxin and TNF-α production while inhibiting the miR-132 target SIRT1. The aberrant production of these cytokines by multiple sclerosis B cells can be normalized by resveratrol. The miR-132-SIRT1 axis controls proinflammatory cytokine secretion by B cells (100). Degeneration of oligodendrocytes (OGDs) and OGD precursors (OPCs) increases with age and is associated with increased inflammatory activity of astrocytes and microglia (101). Genetic inactivation of SIRT1 increases the production of OPCs in the mouse brain, and neo-OPCs differentiate normally to produce fully myelinated oligodendrocytes. Thus, it improved myelin regeneration and delayed paralysis in a mouse model of demyelination injury. SIRT1 inactivation resulted in the upregulation of genes involved in cellular metabolism and growth factor signalling, particularly PDGF receptor alpha (PDGFRα). PDGFRα mediates Oligodendrocyte expansion through its downstream AKT and p38 MAPK signalling molecules (102). SIRT1 inactivation confers anti-inflammatory functions to astrocytes, inhibits the production of pro-inflammatory mediators by myeloid cells and microglia, and promotes the differentiation of oligodendrocyte progenitors. A study in mice revealed that astrocyte-specific SIRT1 knockout (SIRT1^-/-^) inhibited EAE progression, SIRT1^-/-^ astrocytes expressed a series of nuclear factor erythroid-derived 2-like 2 (Nfe2l2) target genes, and Nfe2l2 deficiency changed the beneficial effects of SIRT1^-/-^ astrocytes into deleterious effects (103). DC plays a key role in activating, shaping and preventing the characteristic CNS immune-mediated damage in MS and EAE. The inhibition of Sirt6 diminished the immune response mainly by reducing CXCR4-positive and CXCR4/CCR7-dual-positive DCs in lymph nodes of EAE mice, which was associated with early downregulation of CD40 expression on DCs and elevated levels of the anti-inflammatory cytokine IL-10 (104).

### 5.5 Urinary system: IgA nephropathy and mesangial proliferative glomerulonephritis

IgAN is the most common primary glomerular disease, with a relatively poor prognosis and lack of pathogenesis-based therapy. Compound K, the major absorbable intestinal bacterial metabolite of ginsenosides, ameliorates the inflammatory response in IgA nephropathy. It inhibits NLRP3 inflammatory vesicle activation in renal tissues, macrophages, and bone marrow-derived DCs through NF-κB/NLRP3 on the one hand and enhances the induction of autophagy by increasing SIRT1 expression on the other hand, thereby inhibiting NLRP3 inflammatory vesicles (105). Tris (dibenzylideneacetone) dipalladium (Tris DBA) is a small molecule palladium complex that alleviates immune complex-mediated diseases, particularly IgAN. Treatment of IgAN mice with Tris DBA resulted in significant improvements in renal function, albuminuria, and renal pathology, mainly through the inhibition of NLRP3 inflammatory vesicle initiation signalling and blunting of NLRP3 inflammatory vesicle activation in kidney tissue or cultured macrophages induced by SIRT1 and SIRT3-mediated autophagy (106).

MsPGN is characterized by glomerular thylakoid cell proliferation and extracellular matrix deposition in the thylakoid region, developing into glomerulosclerosis. SRT1720 activation of SIRT1 significantly enhanced the activity of the NRF2/ARE pathway, including promoting the nuclear content and ARE binding capacity of NRF2, increasing the protein levels of two target genes of NRF2, HO-1 and SOD1, which ultimately increased total SOD activity and decreased malondialdehyde levels in renal tissues of experimental anti-Thy 1.1 MsPGN rats and alleviated the pathological process of MsPGN (107).

## 6 Role of SIRTs in systemic autoimmune diseases

### 6.1 Rheumatoid arthritis

RA is an autoimmune disease involving multiple systems, characterized by synovial proliferation, vascular opacification, and bone destruction. The SIRT family is closely associated with the development of RA. The rs3740051, rs7069102, and rs1467568 variants in the SIRT1 gene are associated with RA susceptibility in the Chinese Han population (108). Kara et al. found increased SIRT3-mRNA expression and decreased SIRT2-mRNA expression in RA, whereas both were increased in active RA (109). Fibroblast-like synoviocytes (FLS) are thought to play a central role in the development, progression, and perpetuation of RA. It is capable of producing pro-inflammatory cytokines and proteases that destroy bones and cartilage (110). SIRT1 is involved in inhibiting the activity of FLS and promoting FLS apoptosis. SIRT1 activation induces FLS apoptosis through the activation of cystein-3 and PI3K/Akt signalling pathways. SIRT1 overexpression not only inhibits FLS proliferation, invasion and migration but also reduces the production of pro-inflammatory cytokines, thus alleviating RA synovial inflammation effectively. Zhang et al. found that Overexpression of circ-SIRT1 inhibited the proliferation and induced apoptosis of RA-FLS MH7A cells in addition to reducing IL-1β, IL-6 and TNF-α levels in MH7A cells and suppressing inflammation (111, 112). These effects are associated with NF-κB activation, and SIRT1 reduces p65 protein expression, phosphorylation, and acetylation in RA-FLS to inhibit the NF-κB pathway (113). Also, SIRT1 upregulation inhibits AP-1 and NF-κB activation to reduce COX2 levels in RA-FLS (114). Therefore, silencing of SIRT1 will promote FLS proliferation and adhesion, leading to a poor prognosis of RA (115). However, in the synovial tissue of RA smokers, silencing of SIRT1 reduced FLS proliferation and is accompanied by increased apoptosis of FLS and decreased IL-6 and IL-8 levels (112, 115). Furthermore, SIRT1 promotes FLS invasion and cartilage destruction through an MMP (TIMP1)-dependent mechanism of inhibition. SIRT1 elevation in the RA synovium inhibits TIMP1 expression through deacetylation of TIMP1-associated histones, thereby disrupting transcription factor-specific protein 1 (Sp1) binding to the TIMP1 promoter. In rats with collagen-induced arthritis, SIRT1 depletion promoted TIMP1 expression in synovial tissue and ameliorated cartilage destruction.

Monocyte macrophages are critical for RA pathogenesis. M1 macrophages act as pro-inflammatory mediators in the synovium, whereas M2 macrophages suppress inflammation and promote tissue repair. SIRT1 transgenic (SIRT1-Tg) mice exhibited lower TNF-α and IL-1β expression levels than wild-type mice. Activation of SIRT1/AMPKα signalling exerts anti-inflammatory activity by regulating M1/M2 polarization, thereby reducing the inflammatory response in RA (116). SIRT1 overexpression in adjuvant-induced arthritic rats reshapes the differentiation characteristics of monocytes and inhibits the glycolytic pathway, an effect that is less pronounced in normal cells (117). Alpha-mangiferin (MG) stimulation activates the cholinergic anti-inflammatory pathway, which upregulates SIRT1 signalling, thereby inhibiting M1 polarization through the NF-κB pathway and improving the pathological immune environment in early AIA rats (118). SIRT1 agonists inhibit PMA-induced phosphorylation and nuclear translocation of PU.1, thereby suppressing monocyte-to-macrophage differentiation (119).

Induction of Sirt1 upregulation in RA-FLS using resveratrol may significantly inhibit FLS invasion and reduce the extent of joint damage, which may be achieved by suppressing the expression of MMP-1 and MMP-13. This can also be specifically regulated by SIRT6, which specifically reduces TNF-α-induced MMP-1 production (120). It can also inhibit ROS production and FLS proliferation by activating the SIRT1/NRF2 signalling pathway (121, 122). Additionally, the upregulation of SIRT1-mRNA, which induces apoptosis through the activation of cysteinase-9 and effector cysteinase-3, also inhibits FLS (123).

In summary, an effective way to slow the progression of RA is to promote apoptosis, inhibit FLS proliferation, invasion and migration, and suppress synovial inflammation.SIRT1 produces negative regulatory effects on various pathways, mainly targeting FLS, while also improving the function of other immune cells, such as M1/M2 phenotype switching, to exert an inhibitory effect on inflammation. In addition, SIRT1 also attenuates synovial oxidative damage factors, such as peroxide production. The interaction between oxidative stress and inflammatory response needs to be further investigated and focused on in the future.

### 6.2 Systemic lupus erythematosus

SLE is a multisystemic, chronic, systemic inflammatory disease. The impact of epigenetics on the autoimmune pathogenesis of SLE has been pointed out in many studies in recent years, and epigenetic changes in SLE that have been identified include DNA methylation, histone modifications, and non-coding RNA modifications. Shen et al. found a significant increase in SIRT1-mRNA and protein levels in patients with active lupus nephritis (LN), which is, in part, good evidence (110). Consiglio et al. found that variants in the SIRT1 promoter rs3758391 increased the incidence and activity index of SLE and that the rs3758391 T allele was a risk factor for lupus nephritis (124). Abnormal immune cell activity due to epigenetic alterations is important for the inflammatory immune response to SLE. The development and progression of SLE is associated with dysfunction of the innate and adaptive immune systems, leading to impaired immune tolerance and autoantibody production; T cells, B lymphocytes, and their cytokines are key factors in the pathogenicity of SLE.

SIRT1 low expression alleviates SLE progression. Hu et al. found that transfection of SIRT1-siRNA into MRL/lpr mice interfered with SIRT1-mRNA transcription and translation processes, increased acetylation of cytosolic histones H3 and H4 in CD4^+^ T cells, attenuated autoimmune responses (125). In one study, CD4^+^ T cells isolated from SLE patients transfected with SIRT1-siRNA showed inhibition of DNA methyltransferase 1 (DNMT1) activity; Th17 cytokines, such as IL-17A, IL-22, and IL-23, were positively correlated with disease activity and severity, and a higher proportion of Th17 cells was found in patients with active SLE and high levels of serum IL-17 (126). SIRT1 acts as a regulator of the inhibition of Th17 differentiation, and its reduced expression leads to a lower percentage of Tregs and a higher percentage of Th17 cells. The aryl hydrocarbon receptor (AhR) is a transcription factor involved in various inflammatory diseases, and its ligand is required for CD4^+^ T cell differentiation and maturation into Th17 or Treg. Peripheral blood AhR activation in patients with active SLE (127). Studies on lupus CD4^+^ T cells have shown that UV-B radiation inhibits SIRT1-mRNA and protein expression by activating AhR and suppressing DNMT1 activity in CD4^+^ T cells by binding upstream of the SIRT1 promoter translation initiation site, thus mitigating the progression of the pathological process (128). Conversely, SIRT1 reverses the AhR-induced imbalance between Th17 and Treg populations and promotes IL-17A and IL-22 secretion by CD4^+^ T cells (129). IL-2 is a key Treg cell regulator that regulates FOXP3 expression to maintain immune tolerance. However, SIRT1 deacetylates FOXP3 and suppresses IL-2 transcription by regulating Bclaf1 and nuclear factors in activated T cells, thereby inhibiting Treg proliferation (130). Surprisingly, in some cases, SIRT1 activation also alleviates renal damage in SLE, and RSV has been found to reduce proteinuria and immunoglobulin deposition in lupus nephritis and IgG1 and IgG2a levels in the serum of pristane-induced lupus mice by activating SIRT1 (131). Additionally, resveratrol inhibited the expression of CD69 and CD71 in CD4^+^ T cells and CD4^+^ T cell proliferation, induced CD4^+^ T cell apoptosis, and reduced the ratio of CD4^+^ IFN-γ^+^ Th1 cells and Th1/Th2 cells *in vitro* (110). Immunosuppression caused to some extent by this may account for the protective effect of SIRT1 activators on SLE. High expression of SIRT2 will promote the progression of SLE. Hisada et al. found that the transcription factor inducible cAMP early blocker (ICER) was overexpressed in T cells of SLE patients and lupus-susceptible mice, directly binding to the Sirt2 promoter and promoting its transcription. This led to increased SIRT2 expression in CD4^+^ T cells of patients, which inhibited IL-2 production through the IL-2 gene c-Jun and histone deacetylation (132). Overexpression of SIRT2 increased the proportion of Th17 cells.

Katsuyama et al. provided evidence that elevated CD38 expression in CD8 ^+^ T cells in SLE patients with a high incidence of infection lead to increased acetylated EZH2 through inhibition of SIRT1. Acetylated EZH2 inhibits RUNX3 expression. CD8^+^CD38^+^ T cells can reduce the cytotoxic response of T cells by suppressing the expression of cytotoxicity-related transcription factors (T-bet, RUNX3, and EOMES) through the CD38/NAD/SIRT1/EZH2 axis (133). The abnormal activation of B cells produces excess antibodies leading to systemic inflammation, and enhances their ability to act as antigen-presenting cells (APCs), contributing to T cell activation. Wang et al. transfected mouse B cells BaF3 with SIRT1-shRNA and found that SIRT1 overexpression promoted BaF3 cell proliferation, decreased apoptosis, and increased expression of pro-inflammatory cytokines (IL-6, IL-2, and TNF-α). IL-2 and TNF-α) expression levels were elevated. Additionally, the NF-κB pathway and p65 were significantly activated and phosphorylated, and the expression of B-cell CLL/lymphoma-3 (Bcl-3) was increased (134). In contrast, in MRL-lpr mice, the SIRT1 agonist RSV enhanced the expression of the IgG receptor Fcγ receptor (FcγRIIB), leading to the activation of inhibitory B cell receptors, thereby inducing B cell apoptosis and a significant reduction of B cells in the spleen and bone marrow. After RSV treatment, there was a significant decrease in plasma cells with high FcγRIIB expression, leading to a decrease in serum autoantibody (e.g., IgG1, IgGα) levels and a decrease in immune complex deposition in the kidney (135). This has promising applications in the treatment of SLE, as both T cells and NK cells do not express FcγRIIB, and the development of targeted drugs for this locus can avoid affecting multiple immune cells to a certain extent and reduce the side effects of immunotherapy.

### 6.3 Systemic sclerosis

SSc is a severe autoimmune connective tissue disease characterized by extensive peripheral microangiopathy and progressive cutaneous and visceral fibrosis leading to severe organ dysfunction. In the last few decades, a growing number of studies have explored the contribution of SIRTs to the pathogenesis of systemic sclerosis, highlighting the significant anti-fibrotic effects of SIRT1 and SIRT3. Both SIRT1 and SIRT3 serum levels were significantly reduced in patients with systemic sclerosis compared to those in controls. In systemic sclerosis, a decrease in circulating SIRT1 and SIRT3 levels enhances the severity of cutaneous fibrosis and interstitial lung disease. Reduced serum SIRT1 and SIRT3 levels also correlate with the severity of the microvascular injury, and SIRT3 levels are associated with the development of finger ulcers (136).

SIRT levels are reduced in the tissues of patients with systemic sclerosis, and molecular studies have revealed several mechanisms by which reduced SIRT levels lead to fibrosis, with most attention paid to the regulation of the TGF-β signalling pathway. The activation of SIRTs in cell culture and animal models induces antifibrotic effects, and decreased levels and activity of SIRTs are emerging as pathogenic factors in systemic sclerosis. Restoration of SIRTs expression levels may be therapeutic for patients with systemic sclerosis (137, 138). SIRT1 expression was reduced in patients with systemic sclerosis and TGF-β-dependent experimental fibrosis patients, but this reduction was not sufficient to counteract the excessive activation of TGF-β signalling in systemic sclerosis. However, if SIRT1 is knocked down, TGF-β/SMAD signalling is inhibited, Smad gene activity is diminished, transcription of TGF-β target genes is reduced, and collagen release from fibroblasts is ultimately reduced (139, 140). Resveratrol ameliorates BLM-induced skin inflammation and fibrosis in systemic sclerosis mice by activating SIRT1/mTOR signalling. Amelioration of mTOR has been found in fibroblasts from patients with systemic sclerosis and in skin lesions from BLM-treated mice. Rapamycin is an mTOR-specific inhibitor that significantly inhibits inflammation and fibrosis. SIRT1 activation significantly inhibited the enhanced mTOR expression in skin lesions in BLM-treated mice. However, in BLM-treated cells and mice, resveratrol exerted an inhibitory effect on the expression of inflammatory factors and reduced the collagen levels after mTOR knockdown (138, 141). SIRT3 is associated with increased oxidative stress, leading to fibrosis. TGF-β inhibition of SIRT3 leads to a decrease in SIRT3-dependent deacetylase activity, which results in an inadequate antioxidant response. SIRT3 expression was significantly reduced in systemic sclerosis skin biopsies and transplanted fibroblasts and was inhibited by TGF-ß treatment in normal fibroblasts. The enhancement of cellular SIRT3 by hexafluorine treatment blocked intracellular TGF-β signalling and fibrotic responses and attenuated the activation phenotype of systemic sclerosis fibroblasts, while the accumulation of mitochondrial and cytoplasmic ROS in fibroblasts was reduced (142, 143).

Overall, SIRT1 and SIRT3 have been shown to improve fibrosis by inhibiting TGF-β-induced signalling, and in addition, SIRT1 attenuates the inhibition of the mTOR pathway. Both of these pathways are important for the development of anti-fibrotic therapies. Moreover, the earlier SIRT1 is activated with resveratrol; the better the fibrosis of the skin and other tissues can be improved. Therefore, we predict that SIRT1 may play a preventive therapeutic role in early SSc.

### 6.4 Vasculitis and giant cell arteritis

Anti-neutrophil cytoplasmic antibody-associated vasculitis (AAV) is a systemic autoimmune disease involving hyperactivated neutrophils, inflammatory factors, and ROS. Shimojima et al. investigated the role of Tregs in AAV and showed that SIRT1 levels, to some extent, negatively regulated the AMPK pathway through the mTOR, maintaining the stability of Tregs in AAV (144). Additionally, oxidative stress had been observed to be involved in giant cell arteritis, which was mainly maintained by the enhanced ROS production by immature neutrophils. Levels of both ROS in leukocyte fractions and plasma markers of oxidative stress (lipid peroxidation and total antioxidant capacity) were significantly increased in patients with giant cell arteritis, as compared with those in healthy controls. A significant decrease in SIRT1 expression was found in PBMCs from patients with giant cell arteritis. However, how these alterations contribute to the pathogenesis of giant cell arteritis has not been elucidated (145).

## 7 Treatment strategies and prospects

Over recent years, SIRTs have been increasingly recognized to play an important role in the pathogenesis of innate and adaptive immunity, as well as in autoimmune and inflammatory diseases. In this review, we described the structure of SIRTs and discussed the effects of different intracellular localization of SIRTs on cellular metabolism, differentiation, immunity, apoptosis, oxidative stress, and mitochondrial function based on the important epigenetic modification function of deacetylation of the SIRT family. We further investigated and compared the interconnections and interactions among SIRT family members to elucidate the role of SIRTs in common organ-specific autoimmune diseases (e.g., Graves’ disease, type 1 diabetes, pulmonary fibrosis, IBD, multiple sclerosis) and systemic autoimmune diseases (e.g., SLE, RA, systemic sclerosis). Many of the inflammatory signalling pathways mentioned in the paper have been extensively studied, but research on inhibitors and activators of these inflammation-specific targets has been slow and has not yet been fully used for the treatment of diseases. Resveratrol is one of the more hotly studied SIRT1 agonists. It is considered a potential antioxidant drug for the treatment of various autoimmune diseases as well as for anticancer therapy. Many of these effects are due to modulation by SIRT1 targets, such as PGC-1α and NF-κB. In addition, resveratrol activates AMPK, inhibits cyclooxygenase, and affects the activity of many other enzymes. In T1DM, resveratrol plays a protective role against cellular oxidative stress through the SIRT1-FOXO3a pathway. In IBD, resveratrol treatment restored SIRT1 mRNA levels, inhibited NLRP-3 inflammatory vesicle activation, and ameliorated colitis in mice. In MS, resveratrol treatment reduced the production of pro-inflammatory cytokines such as IL-6 and IL-12/23 p40d in mice. In RA, resveratrol inhibits the activation of the MAPK signalling pathway and IL-1β expression in rat synovial tissue and suppresses the development of arthritis. Activation of PI3K/Akt signalling pathway by excitation-induced apoptosis of FLS. In SLE, resveratrol upregulates FcγRIIB through NF-κB activation to reduce the number of B cells and their antibody production and improve lupus. In addition, several small molecule SIRT1 activators (e.g., SRT2104, SRT2379, SRT3025, etc.) have entered clinical trials. Here, we summarized the current potential therapeutic options for these autoimmune diseases (**Table 2**).

**Table 2:** The studies related to the treatment of autoimmune diseases.

Autoimmune diseases	Animal models	Tissue/Cell types	Substances/Drugs	Pathways	Main mechanisms and effects	References
**GD**	—	PBMC	SIRT1	SIRT1/NF-κβ	The expressions of IL-6, IL-8, TNF-α, and MCP1 were reduced.	(59)
**HT**	—	Thyroid	Th1 cytokines (IL-1α, IFN-γ)	Th1 cytokine/NOX4/ROS/SIRT1/HIF-α	Th1 cytokines caused excessive ROS production by oxidative stress, downregulated SIRT1, upregulated HIF-α, GLUT-1,VEGF-A.	(63)
	—	CD4CD25FOXP3 T cells	SIRT1	SIRT1/FOXP3	SIRT1-mediated aberrant FOXP3 acetylation leaded to reduced FOXP3 expression levels and defective Treg function.	(62)
**T1DM**	DN rats	Kidney	Isoliquiritigenin (ISLQ)	SIRT1/NF-κB	ISLQ inhibited the release of NF-κB, IL-1β, TNF-α and reduced oxidative stress in the kidney.	(146)
	DN/STZ mice	Kidney/Podocyte	miR-34a	P53/miR-34a/SIRT1	Inhibition of P53/miR-34a/SIRT1 axis ameliorated podocyte injury in diabetic nephropathy.	(147)
	CKD rats	Kidney/Mesangial cells (MCs)	RSV	SIRT1/Smad3/TGF-β1	TGF-β1-induced ETM and renal fibrosis were attenuated.	(148)
	DN mice	Kidney/Podocyte	Gardenia jasminoides (GE)	APMK/SIRT1/NF-κB	TNF-α, IL-6 and IL-1β decreased and inhibited the development of DN.	(149)
	DB mice	Kidney/BUMPT cells	Tin-nickelate calcein-1 (STC-1)	AMPK/SIRT3	STC-1 ameliorated renal injury in DN by inhibiting Bnip3 expression through the AMPK/SIRT3 pathway.	(150)
	DN rats	Kidney/MCs	RSV	SIRT1/FOXO1	RSV significantly increased the expression of AdipoR1 by activating FOXO1 in diabetic kidney.	(151)
	—	Kidney/HK-2 cells	Pyrroloquinoline quinine (PQQ)	PI3K/Akt/FOXO3a	PPQ achieved its protective effects through PI3K/Akt/FOXO3a pathway and SIRT3-dependent regulation.	(152)
	DB mice	Kidney/MCs	Flavonoids (FMN)	SIRT1/Nrf2/ARE	FMN upregulated SIRT1 expression to activate the Nrf2/ARE signaling pathway and ameliorated oxidative stress in DN to prevent the progression of renal fibrosis.	(153)
	DN rats	Kidney/MCs	miR-217	SIRT1/HIF-1α	miR-217 promoted inflammation and fibrosis in high glucose cultured rat glomerular thylakoid cells via the SIRT1/HIF-1α signaling pathway.	(154)
**PF**	PF mice	Mesenchymal stem cells (MSCs)	miR-155-5p	SIRT1/AMPK	miR-155-5p inhibition promoted autophagy and ameliorated IPF-MSC senescence by activating the SIRT1/AMPK signaling pathway.	(73)
	PF mice	Lung fibroblasts	AGK2	SIRT2/Smad2/3	Inhibition of SIRT2 alleviated fibroblasts activation and pulmonary fibrosis via Smad2/3 Pathway.	(74)
	PF rats	Lung	Cryptotanshinone (CTS)	TGF-β1/Smad3/STAT3/SIRT3	Fibrosis biomarkers (fibronectin, type I collagen and α-SMA) were significantly downregulated.	(76)
**IBD**	UC mice	Colon/M1 macrophages	Loganin	SIRT1/NF-κB	Loganin inhibited macrophage M1 polarization and regulated SIRT1/NF-κB signaling pathway to reduce ulcerative colitis.	(155)
	Colitis mice	Colon/Tregs	Desmethylisoboldine (NOR)	NAD/SIRT1/SUV39H1/H3K9me3	NOR promoted Treg differentiation and reduced colitis by targeting glycolysis and the subsequent NAD/SIRT1/SUV39H1/H3K9me3 signaling pathway.	(156)
	—	Intestinal epithelial THP-1 cells	Ginsenoside Rk2	SIRT1/ERK/MEK	Ginsenoside Rk2 prevented ulcerative colitis by inactivating the ERK/MEK pathway via SIRT1.	(157)
	UC mice	Colon	Dornithine (Ori)	SIRT1/NF-κB/P53	DSS-induced inflammatory response, oxidative stress and intestinal mucosal apoptosis were Inhibited.	(158)
	UC mice	Intestinal epithelial IEC-6 cells	Atractylenolide III (AT III)	AMPK/SIRT1/PGC-1α	AT III Inhibited the production of pro-inflammatory factors and the reduction of antioxidants and attenuated intestinal epithelial barrier disruption and mitochondrial dysfunction.	(159)
	UC rats	Colon	Umbelliferone(UMB)	TLR4/NF-κB-p65/iNOS;SIRT1/PPARγ	UMB ameliorated acetic acid-induced ulcerative colitis by modulating TLR4/NF-κB-p65/iNOS and SIRT1/PPARγ signaling pathways.	(160)
	Colitis mice	Colon	Chitosan oligosaccharide (COS)	PPARγ/SIRT1/NF-κB	COS down-regulated pro-inflammatory cytokines and up-regulated mucin-2 levels. Activation of PPARγ and SIRT1 inhibited the activation of NF-κB pathway and reduced NO and IL-6 production.	(161)
	Colitis rats	Colon	Cilostazol	cAMP/SIRT1	Cilostazol inhibited NF-κB, Akt and MAPK inflammatory pathways and reduced acetic acid-induced oxidative stress and apoptosis.	(162)
	Colitis rats	Colon	Ligliptin	AMPK/SIRT1/PGC-1α JAK2/STAT3	Ligliptin activated the AMPK-SIRT1-PGC-1α pathway and inhibited the JAK2/STAT3 signaling pathway, downregulated TNF-α, IL-6 and NF-κB p65, and upregulated the anti-inflammatory cytokine IL-10, reducing the severity of colitis.	(163)
	Colitis mice	Colon	Ulva pertusa	NF-κB/Nrf2/SIRT1	Ulva pertusa reduced DNBS-induced tissue damage, inhibited NF-κB-induced inflammatory cascade response, and regulated the expression of p53, Bax, Bcl-2 and cystathione.	(164)
	UC rats	Colon	Engeletin(EMPA)	SIRT1/PI3K/AKT/NF-κB	EMPA counteracted AA-induced UC in rats by modulating the SIRT1/PI3K/AKT/NF-κB inflammatory pathway, normalizing the oxidant/antioxidant balance, and improving the integrity of the colonic barrier.	(165)
**MS**	EAE mice	Spinal Cord/Th17 cells/Tregs	Methylene blue(MB)	AMPK/SIRT1	MB Inhibited pro-inflammatory T-cell responses, significantly reduced clinical scores of EAE, and attenuated pathological damage to the spinal cord.	(96)
	EAE mice	Thymic epithelial cells (TECs)	NAD^+^	PI3K/Akt/mTOR	NAD^+^ stimulation of SIRT1 expression inhibited the PI3K/Akt/mTOR pathway, promoted autophagy in TECs and inhibited the autoimmune state of EAE mice, thereby protecting EAE mice from sustained injury.	(166)
	EAE mice	Th17/Th2 cells	Lipocalin (ADN)	SIRT1/PPARγ/RORγt	ADN Inhibited Th17 differentiation and limited autoimmune CNS inflammation.	(100)
**RA**	—	RA-FLS	GAS5	miR-222-3p/SIRT1	LncRNA GAS5 attenuated RA-FLS proliferation, inflammation, and promoted apoptosis.	(167)
	RA mice	RA-FLS/PBMC/Exos	NEAT1	MicroRNA-23a/MDM2/SIRT6	NEAT1 promoted rheumatoid arthritis.	(168)
	CIA mice	CIA-FLS/Bone/Cartilage	Quercetin (Que)	SIRT1/PGC-1α/NRF1/TFAM	Que Improved impaired mitochondrial function, decreased clinical scores, attenuated synovial inflammatory hyperplasia and bone/chondral destruction, and reduced the secretion of inflammatory factors.	(169)
	AIA rats	M1 macrophages/Th1 cells	Alpha-invertin (MG)	CAP/SIRT1	MG Induced cholinergic anti-inflammatory pathway (CAP) activation, upregulated SIRT1 signaling, inhibited M1 polarization via NF-κB pathway, and improved the pathological immune environment.	(123)
**SLE**	—	CD4^+^T cells/CD1^+^T cells	Ultraviolet B (UVB)	AhR/SIRT1/DNMT1	UVB inhibited the activity of DNMT1 via AhR activation dependent SIRT1 suppression in CD4^+^ T cells.	(129)
	MRL/lpr mice	Splenic CD4^+^ T cells	miR-199a-5p	miR-199a-5p/SIRT1/p53	miR-199a-5p improved lupus symptoms but increased senescence of splenic CD4^+^ T cells.	(170)
	—	CD8CD38^+^T cells	CD38	CD38/NAD/SIRT1/EZH2	CD38 overexpression in CD8^+^ T cells decreased the cytotoxic response of T cells by inhibiting SIRT1 leading to an increase in acetylated EZH2.	(134)
	MRL/lpr mice	B cells	RSV	SIRT1/FcγRIIB/NF-κB	RSV activated SIRT1, enhanced FcγRIIB expression, induced B-cell apoptosis, reduced serum autoantibodies and ameliorated lupus nephritis.	(136)
	—	Monocytes	Pam3CSK4	TLR2/SIRT1/PPAR-γ	Downregulation of SIRT1 enrichment in the PPAR-γ promoter regulated PPAR-γ expression and induced polarization of monocytes toward an M2-like phenotype with increased Arg-1 expression and decreased expression of CD80, NF-κB, IL-1b, IL-6, IL-12, and CCR7.	(171)
	ASLN mice	Kidney	Hauptophanol (HNK)	SIRT1/autophagic axis/NLRP3	Negative regulation of T-cell function and enhanced activation of NLRP3 inflammatory vesicles by reduced SIRT1/autophagy axis to alleviate renal lesions in ASLN mice.	(172)
	LN mice	Splenic lymphocytes	Panax notoginseng saponin (PNS)	SIRT1/FOXO1/MDR1	PNS inhibited SIRT1/FOXO1/MDR1 signaling pathway in lymphocytes and reversed P-gp-mediated steroid resistance in lupus.	(173)
**SSc**	SSc mice	Skin/Fibroblasts	RSV	SIRT1/mTOR	SIRT1 activation inhibited mTOR phosphorylation, suppressed inflammation and fibrosis, and collagen reduction.	(141)
	SSc mice	Fibroblasts	RSV	SIRT1/TGF-β/SMAD	SIRT1 enhanced the pro-fibrotic action of TGF-β with Smad gene activity, elevated transcription of TGF-β target genes, and enhanced release of collagen.	(139)

The vast majority of existing studies have focused on SIRT1, with more limited research on other members of the SIRT family (e.g., the newly identified SIRT7). In addition, studies exploring the relationship between the SIRT family and autoimmune diseases are inadequate. In the future, more detailed interactions and effects of the SIRT family should continue to be refined, with further attention to the substrate changes and gene expression effects induced by the SIRT family. The development and application of specific SIRT family agonists and inhibitors built on this basis should be improved. Detailed mechanistic elaboration of the SIRT family will help to better explore the pathogenesis of autoimmune diseases and thus provide novel therapeutic directions.

## Author contributions

ZT drew the image, built the form and wrote the manuscript, ZJ, JW, and GC collected the literature and revised the manuscript. XY conceived the study and assumed overall responsibility for this work. All authors contributed to the article and approved the submitted version.

## Glossary

**Table:**

adenosine 5’-monophosphate	(AMP)
activated protein kinase	(AMPK)
activator protein 1	(AP-1)
cyclooxygenase-2	(COX2)
dentin matrix acidic phosphoprotein 1	(DMP1)
forkhead box protein O1	(FOXO1)
forkhead box protein O3	(FOXO3)
forkhead box protein O3a	(FOXO3a)
forkhead box protein P3	(FOXP3)
glucose 6-phosphate dehydrogenase	(G6PD)
glutathione SH	(GSH)
hypoxia inducible factor 1, alpha subunit	(HIF-1α)
interleukin-1	(IL-1)
interleukin-1beta	(IL-β)
interleukin-2	(IL-2)
interleukin-6	(IL-6)
interleukin-10	(IL-10)
interleukin-12	(IL-12)
interleukin-17	(IL-17)
interleukin-17f	(IL-17f)
interleukin-18	(IL-18)
interferon gamma	(IFN-γ)
mammalian target of rapamycin	(mTOR)
manganese superoxide dismutase	(MnSOD)
mitogen-activated protein kinase	(MAPK)
matrix metalloproteinase 2	(MMP2)
matrix metalloproteinase 9	(MMP9)
matrix metalloproteinase 13	(MMP13)
monocyte chemotactic protein 1	(MCP-1)
nucleotide-binding oligomerization domain leucine-rich repeat and pyrin domain-containing 3	(NLRP3)
nucleotide-binding oligomerization domain leucine-rich repeat and caspase recruitment domain-containing 4	(NLRC4)
nuclear factor kappa B subunit	(NF-κB)
nuclear erythroid 2-related factor 2	(NRF2)
oxoguanine glycosylase 1	(OGG1)
prostaglandin E2	(PGE2)
peroxisome proliferator-activated receptor γ coactivator 1-alpha	(PGC-1α)
peroxisome proliferator activated receptor alpha	(PPAα)
peroxisome proliferators-activated receptor γ coactivator 1alpha	(PGC-1α)
plasminogen activator inhibitor 1	(PAI-1)
phosphoglycerate mutase 2	(PGAM2)
phosphoinositide-3 kinase	(PI3K)
receptor tyrosine kinase-like orphan receptor gamma t	(RORγt)
signal transducer and activator of transcription 3	(STAT-3)
superoxide dismutase 2	(SOD2).
transforming growth factor-beta 1	(TGF-β1)
tumor necrosis factor-α	(TNF-α)
