# Correction: Sirtuin family in autoimmune diseases

**DOI:** 10.3389/fimmu.2026.1904382

**Published:** 2026-06-24

**Authors:** Zhengjie Tao, Zihan Jin, Jiabiao Wu, Gaojun Cai, Xiaolong Yu

**Affiliations:** 1 Science and Education Section, Wujin Hospital Affiliated with Jiangsu University, Changzhou, Jiangsu, China; 2 Department of Ultrasonics, The Wujin Clinical College of Xuzhou Medical University, Changzhou, Jiangsu, China; 3 Clinical Lab, Changzhou Second People’s Hospital Affiliated to Nanjing Medical University, Changzhou, China; 4 Department of Immunology, Wujin Hospital Affiliated with Jiangsu University, Changzhou, Jiangsu, China; 5 Cardiology, Wujin Hospital Affiliated with Jiangsu University, Changzhou, Jiangsu, China

**Keywords:** sirtuins, autoimmune diseases, immune inflammation, oxidative stress, treatment, review

In section **5 Role of SIRTs in organ-specific autoimmune diseases**, 5.4 *Nervous system: multiple sclerosis*, paragraph 1 the term “gramoxone acetate” was mistakenly used. A correction has been made to the section **5 Role of SIRTs in organ-specific autoimmune diseases**, 5.4 *Nervous system: multiple sclerosis*, paragraph 1:

“Ciriello et al. collected PBMCs from patients with multiple sclerosis treated with glatiramer acetate to determine phosphorylated SIRT1 (p-SIRT1) and H3K9me3 levels.”

The original version of this article has been updated.

